# Nrf2 Deficiency Attenuates Testosterone Efficiency in Ameliorating Mitochondrial Function of the Substantia Nigra in Aged Male Mice

**DOI:** 10.1155/2022/3644318

**Published:** 2022-02-18

**Authors:** Baoliang Ren, Tianyun Zhang, Qiqing Guo, Jing Che, Yunxiao Kang, Rui Cui, Yu Wang, Xiaoming Ji, Guoliang Zhang, Geming Shi

**Affiliations:** ^1^Laboratory of Neurobiology, Hebei Medical University, Shijiazhuang 050017, China; ^2^Department of Neurology, Affiliated Hospital of Hebei University, Baoding 071000, China; ^3^Neuroscience Research Center, Hebei Medical University, Shijiazhuang 050017, China; ^4^Hebei Key Laboratory of Neurodegenerative Disease Mechanism, Hebei Medical University, Shijiazhuang 050017, China

## Abstract

Reduced testosterone level is a common feature of aging in men. Aging, as a risk factor for several neurodegenerative disorders, shows declined mitochondrial function and downregulated mitochondrial biogenesis and mitochondrial dynamics. Mitochondrial biogenesis and mitochondrial dynamics are crucial in maintaining proper mitochondrial function. Supplementation with testosterone is conducive to improving mitochondrial function of males during aging. Nuclear factor erythroid 2-related factor 2 (Nrf2), a regulator of redox homeostasis, is involved in the ameliorative effects of testosterone supplementation upon aging. To explore Nrf2 role in the effects of testosterone supplementation on mitochondrial function during aging, we studied the efficiency of testosterone supplementation in improving mitochondrial function of Nrf2 knockout- (KO-) aged male mice by analyzing the changes of mitochondrial biogenesis and mitochondrial dynamics. It was found that wild-type- (WT-) aged male mice showed low mitochondrial function and expression levels of PGC-1*α*, NRF-1\NRF-2, and TFAM regulating mitochondrial biogenesis, as well as Drp1, Mfn1, and OPA1 controlling mitochondrial dynamics in the substantia nigra (SN). Nrf2 KO aggravated the defects above in SN of aged male mice. Testosterone supplementation to WT-aged male mice significantly ameliorated mitochondrial function and upregulated mitochondrial biogenesis and mitochondrial dynamics, which were not shown in Nrf2 KO-aged male mice due to Nrf2 deficiency. Testosterone deficiency by gonadectomy (GDX) decreased mitochondrial function, downregulated mitochondrial biogenesis, and altered mitochondrial dynamics balance in young male mice. Supplementation with testosterone to Nrf2 KO-GDX mice only ameliorated the alterations above but did not reverse them to sham level. Nrf2 deficiency attenuated testosterone efficiency in ameliorating mitochondrial function in the SN of aged male mice through mitochondrial biogenesis and mitochondrial dynamics to some extent. Activation of Nrf2 might contribute to testosterone-upregulating mitochondrial biogenesis and mitochondrial dynamics in the SN during aging to produce efficient mitochondria for ATP production.

## 1. Introduction

Aging, as a risk factor for several neurodegenerative disorders including Parkinson's disease (PD) and Alzheimer's disease, shows mild to severe mitochondrial dysfunction. Mitochondrial dysfunction, such as the decreased oxidative phosphorylation, the increased reactive oxygen species (ROS), and oxidative damage, is found in aged subjects and subjects with aging-related neurodegeneration [1]. As an energy powerhouse, mitochondria are the main producer of ROS in the cells [2, 3]. While ROS are involved in normal cellular function, the overproduction of ROS disturbs redox homeostasis and contributes to the brain aging and aging-related neurodegeneration by oxidating the biomolecules of neurons [2]. Due to obvious involvement in aging and aging-related neurodegeneration, the mitochondrion is identified as a major target for neuroprotection [4]. The reduction of oxidative damage to mitochondria by enhancing antioxidative capability and the preservation of the normal mitochondrial function via regulating mitochondrial biogenesis or mitochondrial dynamics have been proposed as strategies to mitigate aging and aging-related neurodegenerative disease [3, 5].

Mitochondrial biogenesis is a complex process, during which new mitochondria are formed from preexisting mitochondria through mechanisms involving interaction between the genetic systems of the nucleus and the mitochondria in the cells [4, 6]. Mitochondrial dynamics is another modification process of mitochondrial morphological state through alterations in mitochondrial fission and fusion activities [7]. Both of them are crucial in the maintenance of mitochondrial function to adapt to energy demands and in the regulation of cell metabolism and antioxidant defense [5, 8]. Nuclear factor erythroid 2-related factor 2 (Nrf2) is an important transcription factor controlling the levels of oxygen free radicals. Activation of the Nrf2-antioxidant response elements (ARE) can alleviate pathophysiological processes of neurodegeneration by reducing oxidative stress. Disruption of the Nrf2-ARE pathway results in an increased vulnerability to oxidant neurotoxin [9]. In addition to enhancing redox defense, the Nrf2-ARE pathway facilitates mitochondrial homeostasis and bioenergetics by regulating mitochondrial biogenesis [10] and maintaining balance of mitochondrial dynamics [11–13]. Thus, Nrf2 might be crucial target that affects the efficacy of the intervention strategy imposed on aging and age-related neurodegeneration through manipulating mitochondria.

Previous studies found that testosterone supplementation significantly ameliorates motor behavioral decline, enhances mesodopaminergic activity, and alleviates oxidative damage to the substantia nigra (SN) in aged male rats [14, 15]. Clinical data also showed that the supplementation with testosterone improves both motor symptom and nonmotor symptom of PD men to some extent [16]. The ameliorative effects of testosterone supplementation above might be related to the alteration of mitochondrial biogenesis and mitochondrial dynamics in the process of testosterone treatment [17, 18]. Gonadectomy to adult male rats decreases the gene expression of peroxisome proliferator-activated receptor-*γ* coactivator 1*α* (PGC-1*α*), which is a master regulator of mitochondrial biogenesis, in the hippocampus, and induces a significant reduction of mitochondrial DNA-encoded subunits and a marked elevation of oxidative damage, in the hippocampus and the SN [19, 20]. Orchiectomy upregulates mitochondrial fission protein and downregulates mitochondrial fusion protein in the rat myocardial infarction model, while testosterone replacement reverses these effects of orchiectomy on mitochondrial dynamics in cardiac muscles [17]. Thus, testosterone deficiency is an important factor inducing mitochondrial deficits and brain mitochondrial dysfunction in males [19, 20], the common early events of aging-related neurodegeneration [21]. Supplementation with testosterone in the aging process ameliorates aged-related brain mitochondrial dysfunction [18].

*In vitro* studies revealed that the oxidative stress status seems to play a crucial role in determining a neuroprotective or neurotoxic role of testosterone supplementation [22]. Nrf2, as a regulator of redox homeostasis and mitochondrial homeostasis, might determine the efficacy in the ameliorative effects of testosterone supplementation on aged-related brain mitochondrial dysfunction in males. Thus, to explore roles of Nrf2 in the effects of testosterone supplementation on the mitochondrial dysfunction in the aging process, the present study analyzed the alterations of mitochondrial function, mitochondrial biogenesis, and mitochondrial dynamics in the SN, as well as behavioral and dopaminergic parameters related to the SN, in testosterone-supplemented Nrf2 knockout-aged male mice. Furthermore, for comparison, young male mice were also included in the study to determine the effects of testosterone deficiency on the SN under the condition of Nrf2 knockout without aging factor interference.

## 2. Materials and Methods

### 2.1. Animals and Housing

Male Nrf2 wild-type (WT) ICR mice and Nrf2 knockout (KO) mice were kindly provided by academician Chunyan Li (Neurology Department, Second Hospital of Hebei Medical University). They were genotyped by PCR analysis of tail DNA and housed (3–4 per cage) in an air-conditioned room (22 ± 2°C) on a 12 h light/dark cycle with standard chow and water available ad libitum. All the experimental procedures followed the rules in the “Guidelines for the Care and Use of Mammals in Neuroscience and Behavioral Research” and were approved by the Committee of Institutional Animal Care and Use of Hebei Medical University.

### 2.2. Experiment 1

The aim of Experiment 1 is to investigate whether Nrf2 deficiency affected testosterone efficiency in ameliorating age-related changes related to the SN of male mice. Young KO (KO-young), aged KO (KO-aged), and testosterone propionate- (TP-) supplemented-aged KO (KO-aged-TP) mice were used. For KO-aged-TP mice, the mice received subcutaneous TP injection (1 mg/kg per day) at the age of 21 months and were sacrificed at the age of 23 months. KO-aged mice were subjected to the same treatment using sesame oil. WT mice were treated the same as KO mice and labeled as WT-young, WT-aged, and WT-aged-TP groups, respectively.

### 2.3. Experiment 2

To eliminate aging factors, young male mice were used to explore the effects of testosterone deficiency and testosterone replacement on the parameters analyzed above in Experiment 1. In Experiment 2, KO mice were divided into the sham-operated (KO-sham), the gonadectomized (KO-GDX), and the GDX with TP treatment (KO-GDX-TP) mice. For the GDX mice, anesthetized mice were castrated by the removal of the testes, epididymis, and epididymal fat under aseptic conditions. The sham-operated mice experienced the same surgical treatment except for the bilateral orchiectomies [23]. For KO-GDX-TP mice, the castrated KO mice were subcutaneously injected with TP (1 mg/kg per day) at the age of 3 months and were sacrificed at the age of 5 months. KO-GDX mice experienced the same treatment as KO-GDX-TP mice with sesame oil instead of TP. Young WT mice were processed the same as KO mice and grouped into WT-sham, WT-GDX, and WT-GDX-TP groups.

### 2.4. Open-Field Test

The mice were back or tail-marked and handled for 5 days before the behavioral test. Open-field apparatus (75 × 75 × 30 cm) was placed in a quiet room with illumination of 20 lux. A digital video camera was set up above the arena to record mouse open-field activity. Each mouse was individually placed in the center of the open-field apparatus, and its five-minute open-field activity was recorded for further analysis. Based on a previous study [23], open-field activity related to total path length, exploratory behavior (walking, climbing, rearing, and sniffing), and grooming behavior (latency of grooming, number of grooming, and duration of grooming) was noted and scored in shorthand from the recorded videos by three independent observers (RC, XJ, and GZ) who were blind to the experimental plan. The behavioral data documented by them did not show any interobserver differences (ANOVA, NS).

### 2.5. Footprint Test

Walking gait of mouse was detected via footprint test. The apparatus for footprint test was a tunnel with 10 cm wide × 50 cm long × 10 cm high. The bottom of the tunnel was covered with white paper. During the experiment, each mouse with forepaws and hindpaws dipped in red and black ink, respectively, was placed at the brightly lit end of a tunnel, which was dark at its other end. The mouse walked down the tunnel, having a set of colored footprints on the white paper. After that, the paper with footprints was then removed for later analysis of walking gait defects. Three independent observers (RC, XJ, and GZ) blind to the experimental purpose measured the stride length of forelimb as well as hindlimb, and the overlap of footprints between forelimb and hindlimb at the right or left side. Stride length is the mean of the forelimb or hindlimb strides, and overlap is the mean of the distance between forelimb and hindlimb at the right or left side.

### 2.6. Sample Preparation

For LC-MS/MS, biochemical assay, mitochondrial membrane potential (MMP) detection, quantitative real-time PCR (qPCR), and western blot analysis, mice were sacrificed by decapitation. Their brains were removed quickly, and tissue blocks containing the SN or caudate putamen (CPu) were dissected on an ice-cold plate under stereomicroscopic observation. And then, they were immediately processed for reduced glutathione/oxidized glutathione (GSH/GSSG), malondialdehyde (MDA), H_2_O_2_, ATP, mitochondrial complexes, citrate synthase (CS), and MMP assays or stored at -80°C after freezing in liquid nitrogen for LC-MS/MS, qPCR, or western blotting on the experimental purposes. For immunohistochemistry (IH), mice were anesthetized and perfused transcardially by 4% paraformaldehyde in 0.1 M phosphate buffer (PB, pH 7.4). The tissue blocks containing the SN were postfixed in the same fixative for 4 h at 4°C, dehydrated in graded ethanol, cleared in xylene, and then embedded in paraffin wax. For mitochondrial ultrastructure analysis, mice were anesthetized and perfused transcardially by a fixative containing 2% paraformaldehyde and 1.25% glutaraldehyde in 0.1 M PB. The mouse brain was removed from the cranial cavity and further dissected under a stereomicroscope to collect the SN tissue block. After three washes in PB, the SN blocks were postfixed with 1% osmium tetroxide for 2 h, dehydrated in acetone, and then embedded in Araldite.

### 2.7. LC-MS/MS Assay

Tissue block containing CPu was weighed and homogenized in 80% acetonitrile containing 0.1% formic acid (5 *μ*L) and then processed following a previous study [24]. The homogenates were centrifuged at 14,000g for 10 min at 4°C. The supernatants were collected and used to determine dopamine (DA), 3,4-dihydroxyphenylacetic acid (DOPAC), and homovanillic acid (HVA) levels as previously described [24]. LC separation was performed on an Agilent 1200 LC system (Agilent, Santa Clara, USA) using a Synergi Fusion-RP C18 column (50 mm × 3.0 mm, 4 *μ*m) provided by Phenomenex. MS/MS detection was carried out using a 3200 QTRAP™ LC-MS/MS System (Applied Biosystems, Foster City, CA, USA). The multiple-reaction monitoring mode was used for quantification. The principal validation parameters of the LC-MS/MS are described in a previous study [24].

### 2.8. Biochemical Analysis

#### 2.8.1. GSH/GSSG

The SN tissue blocks were homogenized in a solution provided by the GSH/GSSG kit (Code No. A061-1, Nanjing Jiancheng Bioengineering Institute, China) and then centrifuged at 14,000 × g for 15 min at 4°C. The supernatants from centrifuged homogenates were used to assess the GSH/GSSG ratio following the instructions of the GSH/GSSG kit. GSH and GSSG in the samples were first made to react with 5,5′-dithio-bis-(2-nitrobenzoic acid) (DTNB) to produce a colored reagent. Then, the 2-vinylpyridine reagent was added to the sample supernatant to ensure that GSSG is the only form of glutathione that reacts with the DTNB reagent. Lastly, the GSSG level was subtracted from the total GSH levels to yield the concentration of reduced GSH. GSH and GSSG levels in the SN were assessed at 412 nm by a spectrophotometer.

#### 2.8.2. MDA Levels

MDA was detected based on its reaction with thiobarbituric acid (TBA). For the measurement of MDA levels in the SN, tissue blocks were weighed and homogenized in 10 times (*w*/*v*) ice-cold 0.1 M PB at pH 7.4. The homogenates were centrifuged at 3000 rpm for 10 min, and the supernatant was processed according to the instructions of an MDA kit (Code No. A003-1, Nanjing Jiancheng Bioengineering Institute, China). The absorbance was read at 532 nm by a spectrophotometer.

#### 2.8.3. H_2_O_2_ Levels

For the detection of H_2_O_2_ in the mitochondria of the SN, the mitochondria were isolated using the tissue mitochondria isolation kit (Code C3606, Beyotime Institute of Biotechnology, China). In brief, SN tissue was homogenized in ice-cold buffer (10 mM HEPES, pH 7.5, including 200 mM mannitol, 70 mM sucrose, 1.0 mM EGTA, and 2.0 mg/mL serum albumin) and centrifuged at 1000g at 4°C for 10 min. The supernatant was centrifuged again at 3500g at 4°C for 10 min to collect a mitochondrial pellet. The levels of H_2_O_2_ in the mitochondria were measured spectrophotometrically at 415 nm according to the protocol of the detection kit (Cat. No. AKAO009M, Beijing Boxbio Science & Technology, China).

#### 2.8.4. ATP Levels

For detection of ATP levels, the mitochondria were isolated using the Tissue Mitochondria Isolation Kit (Code C3606, Beyotime Institute of Biotechnology, China). ATP levels were measured in isolated mitochondria using an ATP colorimetric assay kit following the manufacturer's instructions (A095-1-1, Nanjing Jiancheng Biotechnology Institute, China). Total mitochondrial protein samples were incubated with the ATP reaction mixture at 37°C for 30 min and detected at 636 nm using a microplate reader (BioTek Instruments Inc., Highland Park, USA) [25].

#### 2.8.5. Mitochondrial Complex Activities

Mitochondria from the SN tissue block were isolated according to the protocol of the detection kits. The activities of mitochondrial complexes I, II, III, IV, and V were measured spectrophotometrically using detection kits for complex I (Cat. No. AKOP005M) at 340 nm, complex II (Cat. No. AKOP006M) at 605 nm, complex III (Cat. No. AKOP007M) at 550 nm, complex IV at 550 nm (Cat. No. AKOP008M, Beijing Boxbio Science & Technology), or complex V at 340 nm (A089-5-1, Nanjing Jiancheng Institute of Biotechnology, China) according to manufacturer's specifications.

#### 2.8.6. Citrate Synthase Assay

CS activity in the SN was estimated based on the reduction of 5,5′-dithio-bis-(2-nitrobenzoic acid) following the specifications of a citrate synthase kit (Code No. A108, Nanjing Jiancheng Bioengineering Institute, China). The SN tissue blocks were firstly homogenized in 0.01 M ice-cold phosphate buffer saline (PBS, pH 7.4) and centrifuged at 14,000 × g for 15 min at 4°C. And then, the CS activities in the supernatant were measured spectrophotometrically based on the instructions of the citrate synthase kit. Absorption values were obtained spectrophotometrically at 412 nm.

### 2.9. Mitochondrial Membrane Potential Detection

MMP in the SN was detected by the Rhodamine 123 (Rh123) fluorescence method as described previously [26]. The SN tissue block was homogenized in a balanced salt solution and filtered through a nylon mesh screen. The cells were harvested routinely. Following two washes with ice-cold PBS, the cells were incubated in Rh123 solution (10 *μ*g/mL) at 37°C for 30 min. After being washed and resuspended in 1 mL PBS, the cells were immediately analyzed by flow cytometry (excitation/emission wavelengths, 488/534 nm). MMP was determined by analyzing the changes in Rh123 fluorescence intensity.

### 2.10. Quantitative Real-Time PCR Analysis

Total RNA from SN tissue block was isolated according to the manufacturer's protocol using TRizol reagent (Invitrogen, Carlsbad, CA, USA). 1 *μ*g of total RNA was reverse-transcribed using random primers to obtain the first-strand cDNA template. Then, qPCR was performed with 20 *μ*L reaction solution containing 0.8 *μ*L cDNA (diluted 1 : 10), 2 *μ*L-specific primers, and 2x GoTaq® Green Master Mix (Promega, USA). PCR was performed as follows: an initial cycle at 95°C for 10 min, followed by 40 cycles at 95°C for 10 s, 60°C for 20 s, and 72°C for 15 s. The melting curves of the PCR products were analyzed to confirm the specificity of amplification. Gene expression of *PGC-1α*, nuclear respiratory factor 1 (*NRF-1*), *NRF-2*, mitochondrial transcription factor (*TFAM*), mitochondrial fission protein dynamin-related protein (*Drp1*), mitochondrial fusion protein mitofusin 1 (*Mfn1*), or optic atrophy protein 1 (*OPA1*) was analyzed using *GAPDH* as the internal control. For all samples, qPCR was performed in triplicate. Relative quantification was performed using the 2^-*ΔΔ*Ct^ method. The sets of primers were as follows: *PGC-1α* (5′-GAAAGGGCCAAACAGAGAGA-3′ and 5′-GTAAATCACACGGCGCTCTT-3′), *NRF-1* (5′-TGGAGTCCAAGATGCTAATG-3′ and 5′-AGAGCTCCATGCTACTGTTC-3′), *NRF-2* (5′-TCAGTGACTCGGAAATGGAG-3′ and 5′-TTCACGCATAGGAGCACTGT-3′), *TFAM* (5′-CAGGAGGCAAAGGATGATTC-3′ and 5′-CCAAGACTTCATTTCATTGTCG-3′), *Drp1* (5′-CAGGAATTGTTACGGTTCCCTAA-3′ and 5′-CCTGAATTAACTTGTCCCGTGA-3′), *Mfn1* (5′-AACTTGATCGAATAGCATCCGAG-3′ and 5′-GCATTGCATTGATGACAGAGC-3′), *OPA1* (5′-GATGACACGCTCTCCAGTGA-3′ and 5′-TCGGGGCTAACAGTACAACC-3′), and *GAPDH* (5′-ACTCTTCCACCTTCGATGCC-3′ and 5′-TCTTGCTCAGTGTCCTTGCT-3′). Accession numbers of the genes for primers are listed in Table S1.

### 2.11. Analysis of mtDNA Copy Number

Total DNA was extracted from the SN tissue blocks using an Animal Tissue Genomic DNA kit (ZP307-2, ZOMANBIO, China) according to the manufacturer's protocol. Mitochondrial DNA (mtDNA) copy number was determined by quantifying *16S rRNA* from mtDNA and nuclear-encoded hexokinase 2 (*HK2*) gene expression via qPCR. qPCR was carried out with 1 *μ*L of sample DNA (diluted 1 : 10), 2 *μ*L of each specific primer, and 2x All-in-OneTM qPCR Mix (GeneCopoeia Inc., USA) in a final volume of 10 *μ*L. The primers for *16S rRNA* and *HK2* were used as follows: *16S rRNA* (5′-CCGCAAGGGAAAGATGAAAGAC-3′ and 5′-TCGTTTGGTTTCGGGGTTTC-3′) and *HK2* (5′-GCCAGCCTCTCCTGATTTTAGTGT-3′ and 5′-GGGAACACAAAAGACCTCTTCTGG-3′). Accession numbers of the genes for primers are listed in Table S1. PCR was performed as follows: an initial cycle at 95°C for 15 min, followed by 40 cycles of 95°C for 10 s, 60°C for 20 s, and 72°C for 20 s. The melting curves of the PCR products were analyzed to confirm the specificity of amplification. qPCR was performed in triplicate. Relative mtDNA copy number was calculated by the ratio between *16S rRNA* and *HK2* genes using the 2^-*ΔΔ*Ct^ method.

### 2.12. Western Blot Analysis

The SN or CPu tissue block was homogenized in radioimmunoprecipitation assay buffer containing 1% Triton X-100, 0.1% SDS, 0.5% sodium deoxycholate, and protease inhibitors (phenylmethanesulfonyl fluoride 100 *μ*g/mL, aprotinin 30 *μ*g/mL, and sodium orthovanadate 1 mM) and sonicated for 4 × 10 s. After centrifugation at 12,000g for 20 min at 4°C, the supernatant was collected and stored at −80°C for detection of target protein based on the study needs. The pellets were homogenized in ice-cold lysis buffer (20 mM HEPES, pH 7.9, 400 mM NaCl, 1 mM EDTA, and 0.1 mM EGTA) for 15 min. The homogenate was centrifuged at 12,000g for 10 min at 4°C, and the supernatant was collected for detection of nuclear Nrf2 protein. Samples from the supernatant were diluted in 5x sample buffer (50 mM Tris, pH 6.8, 2% SDS, 10% glycerol, 0.1% bromophenol blue, and 5% *β*-mercaptoethanol) and heated for 5 min at 95°C before SDS-PAGE on a 10% gel and transferred to a PVDF membrane (Millipore). The membrane was incubated for 2 h with 5% nonfat dry milk in Tris-buffered saline (TBS) containing 0.05% Tween-20 (TBST). After being rinsed thrice with TBST, the membrane was incubated overnight with rabbit anti-tyrosine hydroxylase (TH) (1 : 10,000, Abcam), rabbit anti-dopamine transporter (DAT) (1 : 1000, Sigma), rabbit anti-PGC-1*α* (1 : 1000, Abcam), rabbit anti-NRF-1 (1 : 1000, ABclonal), rabbit anti-NRF-2 (1 : 1000, ABclonal), rabbit anti-TFAM (1 : 1000, GeneTex), rabbit anti-Drp1 (1 : 1000, Cell Signaling Technology), rabbit anti-pDrp1-S616 (1 : 1000, Affinity), rabbit anti-Mfn1 (1 : 1000, arigo), rabbit anti-OPA1 (1 : 1000, GeneTex), mouse anti-Nrf2 (1 : 500, Santa Cruz Biotechnology), rabbit anti-heme oxygenase 1 (HO-1, 1 : 300, Affinity), rabbit anti-*β*-actin (1 : 10,000, ABclonal), or rabbit anti-H3 (1 : 1000, Arigo) antibody at 4°C according to the study purposes. After three washes, the membrane was incubated for 1 h in IRDye® 800-conjugated goat anti-rabbit (1 : 10,000; Rockland) or anti-mouse (1 : 5,000; Rockland) second antibody. The bands were scanned by an Odyssey infrared scanner (LI-COR Biosciences). The densitometry values of the individual protein were normalized with respect to those of *β*-actin or H3, which was used as the endogenous control. For detecting the oxidation of mitochondrial proteins, mouse anti-3-nitrotyrosine (3-NT) antibody (1 : 1000, Santa Cruz Biotechnology) or rabbit anti-VDAC (1 : 1000, ABclonal) was used to incubate the PVDF containing the electroblotted proteins from isolated mitochondrial fractions. After performing the same western blot procedures as described before, 3-NT densitometry values were normalized with those of VDAC that was used as mitochondrial endogenous control.

### 2.13. Immunohistochemistry

5 *μ*m coronal sections sliced from paraffin-embedded SN tissue blocks were mounted on the slides. After deparaffinization and hydration, the sections were processed for antigen retrieval, inactivation of endogenous peroxidase activity, and incubation in normal serum. Subsequently, the sections were incubated with mouse anti-3-nitrotyrosine (3-NT) antibody (1 : 100, Santa Cruz Biotechnology) overnight at 4°C. After washing, the sections were incubated with biotinylated goat anti-mouse IgG (1 : 500) for 2 h at room temperature. Following incubation at room temperature in horseradish peroxidase-conjugated streptavidin (1 : 500) for 1 h, the sections were stained for 5 min in a solution containing 0.05% diaminobenzidine and 0.03% H_2_O_2_ in 0.05 M Tris-HCl buffer (pH 7.6). A computer-assisted image analysis system (Image-Pro Plus 6.0) was used to measure the average optical density (AOD) and the number of 3-NT immunoreactive (3-NT-ir) positive cells in the SN.

### 2.14. Mitochondrial Ultrastructure Analysis

Ultrathin sections (70 nm) were obtained with a microtome (UC-7, Leica, Austria). After staining with uranyl acetate (10 min) and lead citrate (5 min), the sections were examined under a transmission electron microscope (Hitachi HT7800, Japan) operated at 80 kV. For the electron microscopy (EM) image analyses, the mitochondrial number was counted using Image-Pro Plus 6.0 (Media Cybernetics, USA) at ×3000 magnification, and the mitochondrial ultrastructure was analyzed at ×25,000 magnification.

### 2.15. Statistics

The data are presented as the mean ± SD. All the data were analyzed by a two-way ANOVA. If the two-way ANOVA was significant, we performed planned comparisons using one-way analysis of variance (one-way ANOVA) for the comparison of treatment effect among same genotype or using Student's *t*-test for the comparison of genotype effect among same treatment. For one-way ANOVA, Levene's test was applied to test of homogeneity of variance. If homogeneity of variance is equal (the significance of Levene's test is greater than 0.05), go to the homogeneity of variance tests (*F*-statistic), where *P* < 0.05, followed by Tukey's honestly significant difference (Tukey's HSD) post hoc test for multiple comparisons. If homogeneity of variance is unequal (the significance of Levene's test is less than 0.05), go to the test of Welch's *F* test (*F*′-statistic), where *P* < 0.05; the post hoc test between groups were done using the Games-Howell procedure. *P* < 0.05 was considered statistically significant.

## 3. Results

### 3.1. Nrf2 Deficiency Attenuated Testosterone Efficiency in Improving Open-Field Activity and Walking Gait of Aged Male Mice

**Figure 1:**
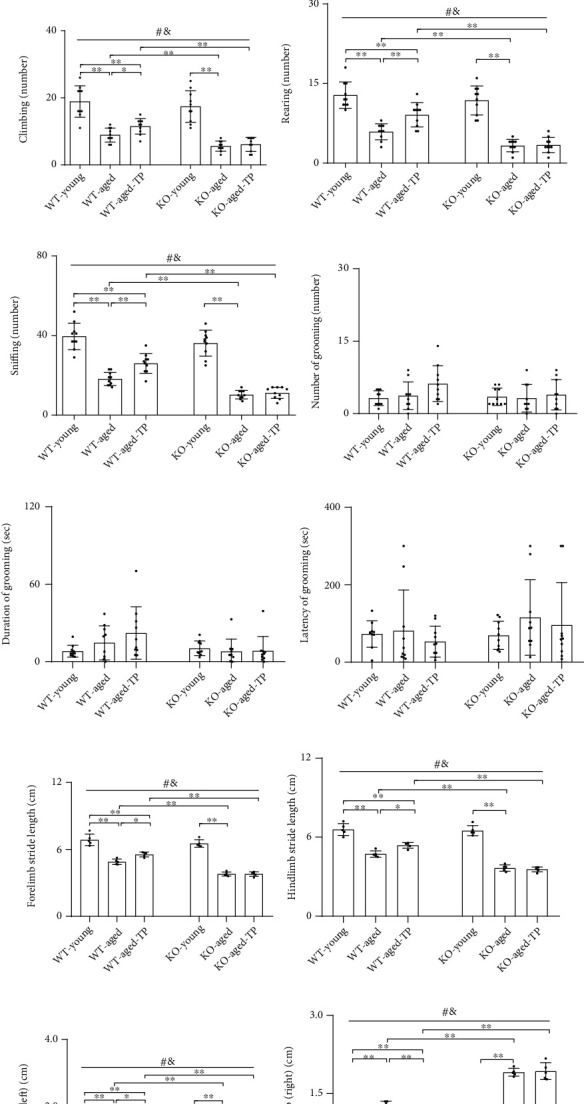
Effects of TP supplementation on open-field activity and walking gait of Nrf2 KO-aged male mice: (a) total path length, (b) walking, (c) climbing, (d) rearing, (e) sniffing, (f) number of grooming, (g) duration of grooming, (h) latency of grooming, (i) forelimb stride length, (j) hindlimb stride length, (k) overlap of left footprints, and (l) overlap of right footprints. Data were presented as mean ± SD; *n* = 10 for open-field test; *n* = 5 for footprint test. ^#^*P* < 0.05 main effect of genotype by two-way ANOVA; ^&^*P* < 0.05 main effect of treatment by two-way ANOVA. ^∗^*P* < 0.05 and ^∗∗^*P* < 0.01.

We first performed open-field test and footprint test to observe the behavioral changes of experimental mice among KO-young, KO-aged, and KO-aged-TP, as well as WT-young, WT-aged, and WT-aged-TP groups. Analysis to them revealed declined total path length, walking, climbing, rearing, and sniffing, as well as decreased stride length and increased overlap of footprints in WT-aged mice and KO-aged mice compared with corresponding control of their own (*P* < 0.01, Figures 1(a)–1(e) and 1(i)–1(l)). Further reduction in total path length, walking, climbing, rearing, sniffing, and stride length, as well as further increment in overlap of footprints, was found in KO-aged mice compared with WT-aged mice (*P* < 0.01). The male mice between WT-young and KO-young groups did not show significant difference in above behavioral parameters. TP supplementation to WT-aged mice significantly increased their total path length, walking, climbing, rearing, sniffing, and stride length and decreased overlap of footprints. These effects by TP were not shown in KO-aged-TP mice. There was no significant intergroup difference among KO-young, KO-aged, KO-aged-TP, WT-young, WT-aged, and WT-aged-TP mice in grooming behavior (Figures 1(f)–1(h)). TP supplementation ameliorated open-field activity and walking gait in WT-aged male mice, but not in KO-aged male mice.

### 3.2. Nrf2 Deficiency Attenuated Testosterone Efficiency in Enhancing Nigrostriatal Dopaminergic Activity of Aged Male Mice

**Figure 2:**
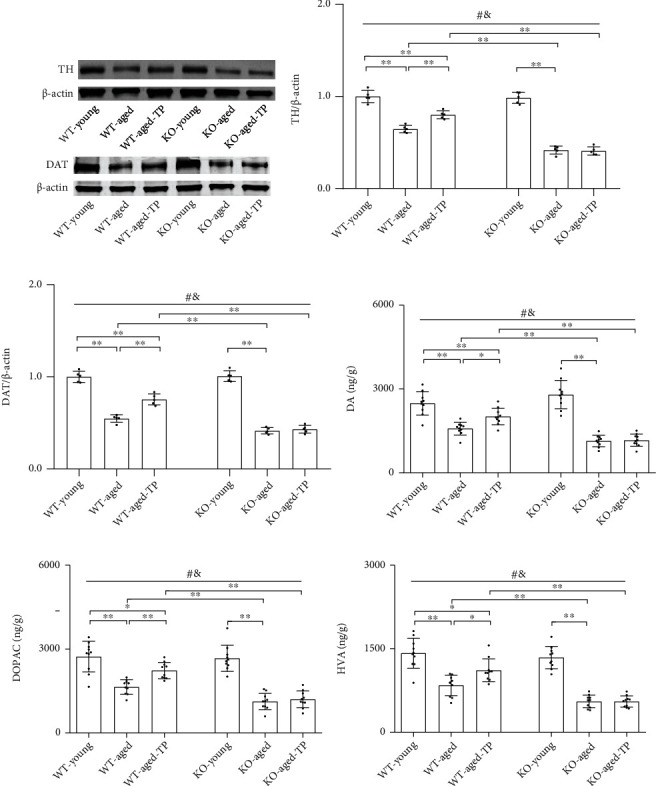
Effects of TP supplementation on dopaminergic activity in the caudate putamen of Nrf2 KO-aged male mice. (a, b) TH and (a, c) DAT were detected by immunoblotting. (d) DA, (e) DOPAC, and (f) HVA were measured by LC-MS/MS assay. Data were presented as mean ± SD; *n* = 5 for TH and DAT; *n* = 10 for DA, DOPAC, and HVA. ^#^*P* < 0.05 main effect of genotype by two-way ANOVA; ^&^*P* < 0.05 main effect of treatment by two-way ANOVA. ^∗^*P* < 0.05 and ^∗∗^*P* < 0.01.

The SN is a brain region rich in dopaminergic neurons. It controls motor behavior and exploratory behavior through a target region in the CPu [27–29]. Thus, we analyzed the altered status of nigrodopaminergic neurons of aged experimental mice under the Nrf2 deficiency by detecting TH and DAT expression, as well as dopaminergic neurochemical content in the CPu. The levels of TH and DAT, as well as DA, DOPAC, and HVA in the CPu, were lower in aged male mice of both genotypes than their corresponding young control mice (*P* < 0.01), and they were much lower in KO-aged mice than in WT-aged mice (*P* < 0.01, Figures 2(a)–2(f)). There was no difference between WT-young mice and KO-young male mice in the levels of TH and DAT, as well as DA, DOPAC, and HVA in the CPu. Administration of TP significantly increased the expression levels of TH and DAT, as well as DA, DOPAC, and HVA in the CPu of WT-aged mice. Increased parameters above by TP were not observed in the CPu of KO-aged-TP mice. TP supplementation increased TH and DAT expression, as well as dopaminergic neurochemical content in the CPu of WT-aged male mice, not in KO-aged male mice.

### 3.3. Nrf2 Deficiency Attenuated Testosterone Efficiency in Ameliorating Oxidative Balance in the SN of Aged Male Mice

**Figure 3:**
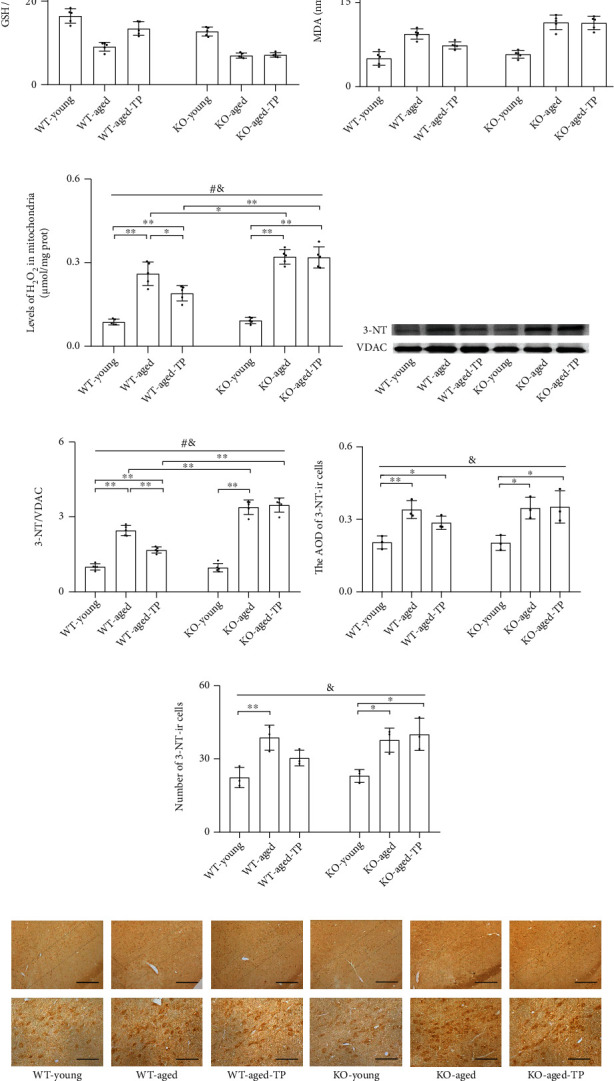
Effects of TP supplementation on oxidative balance in the substantia nigra of Nrf2 KO-aged male mice. (a) GSH/GSSG, (b) MDA, and (c) mitochondrial H_2_O_2_ were assessed by spectrophotometry. (d, e) Mitochondrial 3-NT was measured by immunoblotting; (f–h) 3-NT in the SN was detected by immunohistochemistry. Data were presented as mean ± SD; *n* = 5 for GSH/GSSG, MDA, mitochondrial H_2_O_2_, and mitochondrial 3-NT; *n* = 3 for 3-NT immunohistochemistry. Scale bars = 50 *μ*m (lower panel); scale bars = 200 *μ*m (upper panel). ^#^*P* < 0.05 main effect of genotype by two-way ANOVA; ^&^*P* < 0.05 main effect of treatment by two-way ANOVA. ^∗^*P* < 0.05 and ^∗∗^*P* < 0.01.

Oxidative balance is critically involved in the aging process; therefore, in the SN, we next detected important parameters related to oxidative balance, i.e., GSH/GSSG ratio (a major biomarker of redox status in biological systems), MDA (a marker of ROS-mediated cell membrane damage), and 3-NT (an oxidative stress biomarker of protein nitration) in the tissue, as well as the levels of H_2_O_2_ and 3-NT in the mitochondria. Significantly low GSH/GSSG ratio and high MDA and mitochondrial H_2_O_2_ levels in the SN, as well as increased 3-NT levels of the cells and mitochondria in the SN, were found in aged male mice of two genotypes compared with corresponding young male mice (Figures 3(a)–3(h)). Nrf2 KO further lowered GSH/GSSG ratio and elevated MDA levels as well as mitochondrial H_2_O_2_ and 3-NT levels of aged male mice. Compared with WT-young mice, KO-young mice showed a reduced GSH/GSSG ratio in the SN (*P* < 0.01). Supplementation with TP significantly increased GSH/GSSG ratio and decreased MDA levels as well as mitochondrial H_2_O_2_ and 3-NT levels of WT-aged male mice. A slight, nonsignificant reduction of 3-NT levels of cells was detected in the SN of WT-aged-TP mice relative to WT-aged mice (Figures 3(f)–3(h)). Increased GSH/GSSG ratio as well as decreased MDA, mitochondrial H_2_O_2_, and 3-NT by TP was not shown in KO-aged-TP mice. TP supplementation ameliorated oxidative balance in the SN of WT-aged male mice, not in KO-aged male mice.

### 3.4. Nrf2 Deficiency Attenuated Testosterone Efficiency in Ameliorating Mitochondrial Function in the SN of Aged Male Mice

**Figure 4:**
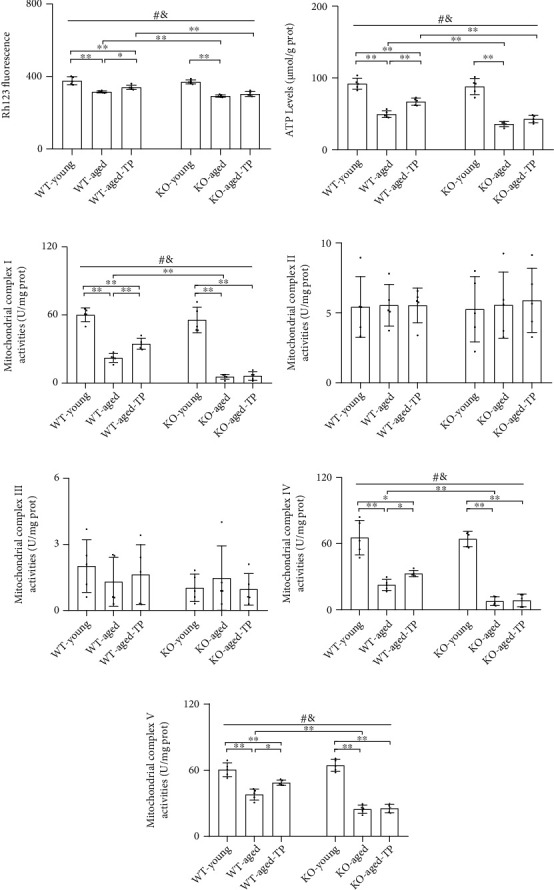
Effects of TP supplementation on mitochondrial function in the substantia nigra of Nrf2 KO-aged male mice. (a) Mitochondrial membrane potential was revealed using the Rh123 fluorescence method. (b) Mitochondrial ATP level, (c) mitochondrial complex I activity, (d) mitochondrial complex II activity, (e) mitochondrial complex III activity, (f) mitochondrial complex IV activity, and (g) mitochondrial complex V activity were revealed by spectrophotometry. Data were presented as mean ± SD; *n* = 5. ^#^*P* < 0.05 main effect of genotype by two-way ANOVA; ^&^*P* < 0.05 main effect of treatment by two-way ANOVA. ^∗^*P* < 0.05 and ^∗∗^*P* < 0.01.

As an early event in aging and age-related neurodegenerative diseases, mitochondrial dysfunction leads to insufficient energy and excessive ROS. Healthy neuronal status depends on proper energy supply and oxidative balance. So we further assessed the effects of Nrf2 deficiency on mitochondrial function in the SN of aged male mice during TP supplementation by measuring MMP, ATP levels, and mitochondrial complex activities. Decreased MMP and mitochondrial ATP levels, as well as the activities of mitochondrial complexes I, IV, and V, were detected in the SN of aged male mice of two genotypes compared with corresponding young male mice (*P* < 0.01, Figures 4(a)–4(c), 4(f), and 4(g)). Nrf2 KO further decreased them in the SN of aged male mice (*P* < 0.01). No significant difference in MMP and mitochondrial ATP levels, as well as activities of mitochondrial complexes I, IV, and V, was found between WT-young and KO-young mice. Supplementation with TP increased MMP and mitochondrial ATP levels, as well as the activities of mitochondrial complexes I, IV, and V in the SN of WT-aged male mice. Increased MMP and mitochondrial ATP levels, as well as the activities of mitochondrial complexes I, IV, and V, were not shown in the SN of KO-aged-TP mice. There was no significant intergroup difference among KO-young, KO-aged, KO-aged-TP, WT-young, WT-aged, and WT-aged-TP mice in the activities of mitochondrial complexes II and III (Figures 4(d) and 4(e)). TP supplementation ameliorated mitochondrial function in the SN of WT-aged male mice, but not in KO-aged male mice.

### 3.5. Nrf2 Deficiency Attenuated Testosterone Efficiency in Increasing PGC-1*α* and Its Downstream Target Expression in the SN of Aged Male Mice

**Figure 5:**
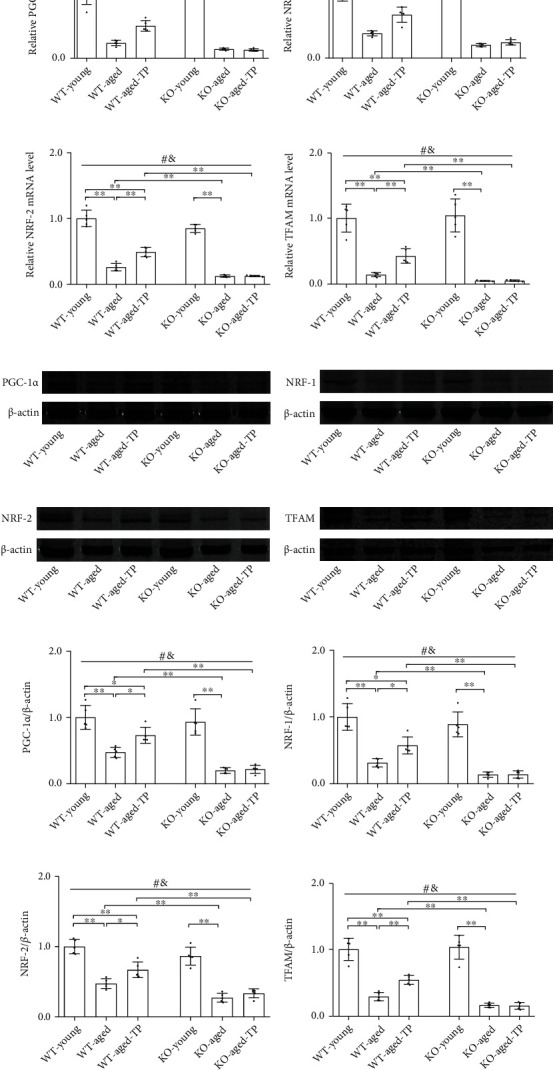
Effects of TP supplementation on mitochondrial biogenesis in the substantia nigra of Nrf2 KO-aged male mice. (a) *PGC-1α*, (b) *NRF-1*, (c) *NRF-2*, and (d) *TFAM* mRNAs were revealed by qPCR. (e, i) PGC-1*α*, (f, j) NRF-1, (g, k) NRF-2, and (h, l) TFAM proteins were detected by immunoblotting. Data were presented as mean ± SD; *n* = 5. ^#^*P* < 0.05 main effect of genotype by two-way ANOVA; ^&^*P* < 0.05 main effect of treatment by two-way ANOVA. ^∗^*P* < 0.05 and ^∗∗^*P* < 0.01.

Based on the decreased mitochondrial biogenesis signaling in aging process, the improved mitochondrial function in aging via the induction of mitochondrial biogenesis [30], and the above-found effects of Nrf2 deficiency on mitochondrial function of aged male mice during TP supplementation, we next examined the altered expression of key inducer and effectors of mitochondrial biogenesis, namely, PGC-1*α*, NRF-1, NRF-2, and TFAM in the SN of experimental mice. Aged male mice of both genotypes showed decreased mRNA levels of *PGC-1α*, *NRF-1*, *NRF-2*, and *TFAM* in the SN relative to respective young control (*P* < 0.01, Figures 5(a)–5(d)). Nrf2 KO further reduced their mRNA levels in the SN of aged male mice (*P* < 0.01). *PGC-1α*, *NRF-1*, *NRF-2*, and *TFAM* mRNA levels were not significantly different in the SN of KO-young mice relative to WT-young mice. Supplementation with TP significantly increased *PGC-1α*, *NRF-1*, *NRF-2*, and *TFAM* mRNA levels in the SN of WT-aged male mice. There was no difference between KO-aged mice and KO-aged-TP mice in the expression levels of *PGC-1α* and its downstream targets. Immunoblotting data from PGC-1*α*, NRF-1, NRF-2, and TFAM agreed with their mRNA changes (Figures 5(e)–5(l)). TP supplementation increased the expression levels of PGC-1*α* and its downstream targets in the SN of WT-aged male mice, but not in KO-aged male mice.

### 3.6. Nrf2 Deficiency Attenuated Testosterone Efficiency in Increasing Mitochondrial Content in the SN of Aged Male Mice

**Figure 6:**
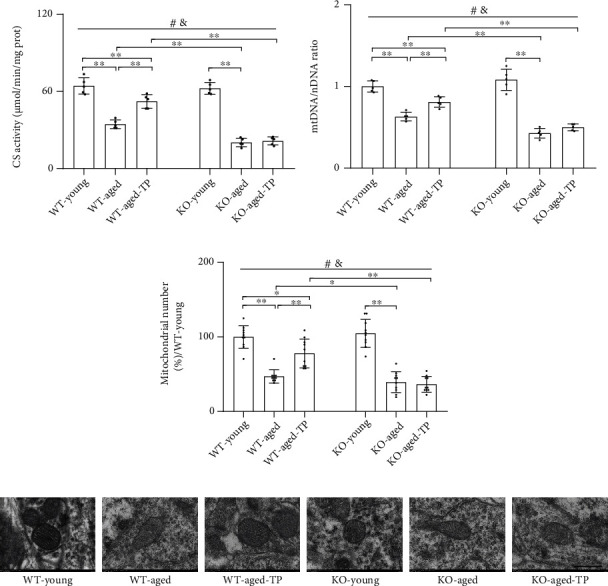
Effects of TP supplementation on mitochondrial content in the substantia nigra of Nrf2 KO-aged male mice. (a) CS activity was assessed by spectrophotometry. (b) mtDNA/nDNA was detected by qPCR. (c) Mitochondrial number was counted using Image-Pro Plus 6.0 by an electron microscope. (d) Mitochondrial ultrastructure images were taken by an electron microscope. Data were presented as mean ± SD; *n* = 5 for CS activity and mtDNA/nDNA; *n* = 2 for an electron microscope (mitochondrial number from an analysis of 5 images per sample from each group). Scale bar = 500 nm. ^#^*P* < 0.05 main effect of genotype by two-way ANOVA; ^&^*P* < 0.05 main effect of treatment by two-way ANOVA. ^∗^*P* < 0.05 and ^∗∗^*P* < 0.01.

Since stimulation of mitochondrial biogenesis is accompanied by increased mitochondrial content, so following, we investigated the effects of Nrf2 deficiency on mitochondrial content in the SN of aged mice supplemented with TP through detecting CS activity (a mitochondrial matrix enzyme), mtDNA copy number, and mitochondrial number. CS activity, mtDNA copy number, and mitochondrial number were reduced in the SN of aged male mice of both genotypes relative to their corresponding young control (*P* < 0.01, Figures 6(a)–6(c)). KO-aged male mice showed much lower CS activity mtDNA copy number and mitochondrial number in the SN than WT-aged male mice. There is no significant difference in CS activity, mtDNA copy number, and mitochondrial number in the SN between KO-young male mice and WT-young male mice. Supplementation with TP increased CS activity, mtDNA copy number, and mitochondrial number in the SN of WT-aged male mice (*P* < 0.01). Increased CS activity, mtDNA copy number, and mitochondrial number by TP were not found in KO-aged-TP mice. In addition, there were striking differences in the mitochondrial ultrastructure among the experimental groups. Compared with mitochondria from the SN of WT-young mice, which presented a normal mitochondrial structure with clear cristae, most mitochondria from the WT-aged or KO-aged male mice showed disorganized cristae. Supplementation with TP to WT-aged male mice improved the ultrastructural alterations of mitochondrial cristae in the SN, which was not observed in KO-aged male mice (Figure 6(d)). TP supplementation increased mitochondrial content in the SN of WT-aged male mice, but not in KO-aged male mice.

### 3.7. Nrf2 Deficiency Attenuated Testosterone Efficiency in Regulating Levels of Drp1, Mfn1, and OPA1 in the SN of Aged Male Mice

**Figure 7:**
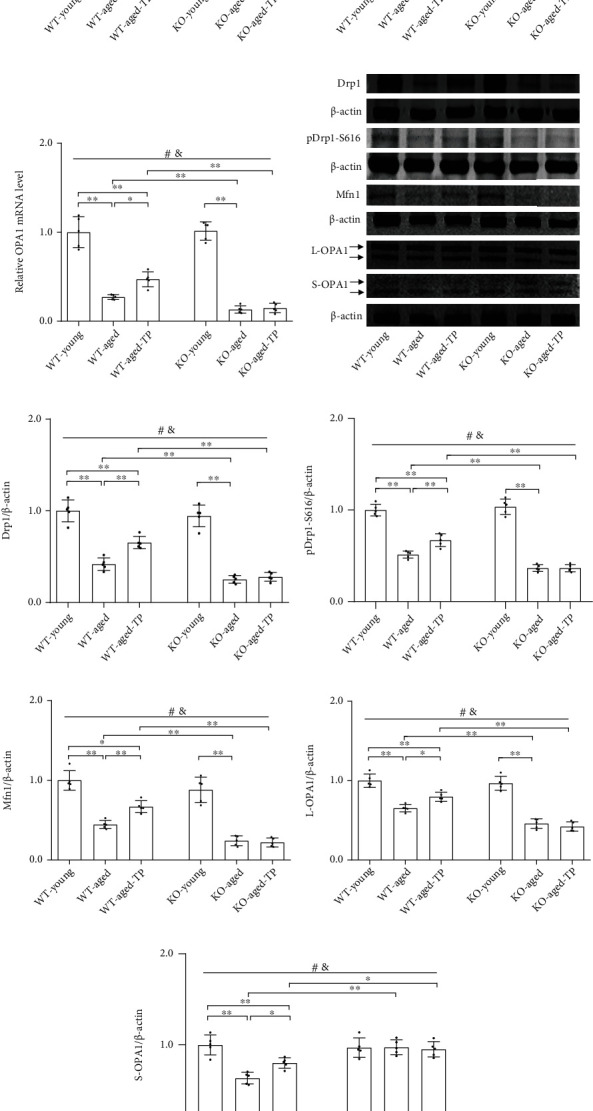
Effects of TP supplementation on mitochondrial dynamics in the substantia nigra of Nrf2 KO-aged male mice. (a) *Drp1*, (b) *Mfn1*, and (c) *OPA1* mRNA were revealed by qPCR. (d, e) Drp1, (d, f) pDrp1-S616, (d, g) Mfn1, (d, h) L-OPA1, and (d, i) S-OPA1 proteins were detected by immunoblotting. Data were presented as mean ± SD; *n* = 5. ^#^*P* < 0.05 main effect of genotype by two-way ANOVA; ^&^*P* < 0.05 main effect of treatment by two-way ANOVA. ^∗^*P* < 0.05 and ^∗∗^*P* < 0.01.

Mitochondrial dynamics take part in maintaining mitochondrial function, and abnormal mitochondrial dynamics are shown in aging and age-related neurodegenerative conditions. Therefore, we analyzed the alterations in the levels of Drp1 and its phosphorylation (pDrp1-S616), Mfn1, and two OPA1 forms (long OPA1: L-OPA1 and short OPA1: S-OPA1) in the SN of experimental mice, which are involved in regulating mitochondrial dynamics. Aged male mice of both genotypes showed reduced mRNA levels of *Drp1*, *Mfn1*, and *OPA1* in the SN compared with respective young control (*P* < 0.01, Figures 7(a)–7(c)). Nrf2 KO further reduced their mRNA levels in the SN of aged male mice. There was no significant difference between WT-young and KO-young mice in mRNA levels of *Drp1*, *Mfn1*, and *OPA1* in the SN. Supplementation with TP increased *Drp1*, *Mfn1*, and *OPA1* mRNA levels in the SN of WT-aged male mice. Increased mRNA levels of *Drp1*, *Mfn1*, and *OPA1* by TP were not detected in the SN of KO-aged-TP mice. Immunoblotting data showed the levels of Drp1, pDrp1-S616, Mfn1, L-OPA1, and S-OPA1 were significantly reduced in the SN of WT-aged mice relative to WT-young mice (Figures 7(d)–7(i)). They were much lower in KO-aged mice than in WT-aged mice, except for S-OPA1 (Figure 7(i)). Significantly increased levels of Drp1, pDrp1-S616, Mfn1, L-OPA1, and S-OPA1 were present in the SN of WT-aged-TP mice compared with WT-aged mice and were not found in KO-aged-TP mice relative to KO-aged mice. TP supplementation increased the levels of Drp1, pDrp1-S616, Mfn1, and two OPA1 forms in the SN of WT-aged male mice, but not in Nrf2 KO-aged male mice.

### 3.8. The Effects of TP Supplementation on Nrf2 in the SN of Aged Male Mice

**Figure 8:**
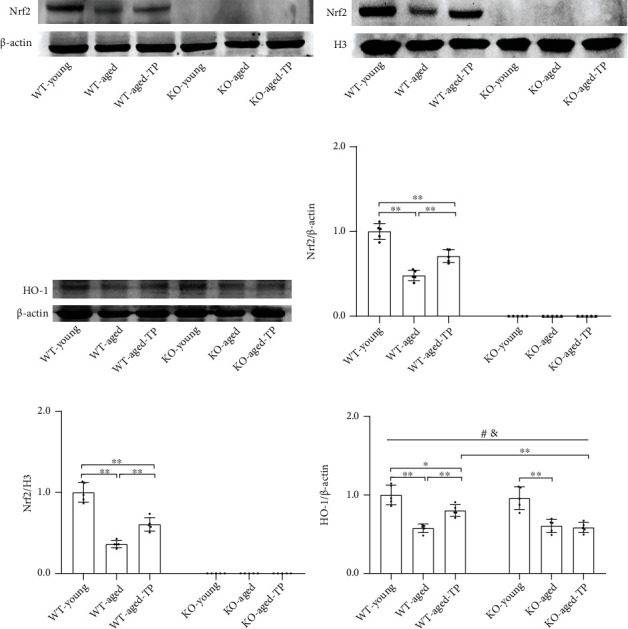
Effects of TP supplementation on Nrf2 in the substantia nigra of aged male mice. (a, d) Nrf2 in the SN, (b, e) Nrf2 in nucleus fraction, and (c, f) HO-1 protein in the SN were detected by immunoblotting. Data were presented as mean ± SD; *n* = 5. ^#^*P* < 0.05 main effect of genotype by two-way ANOVA; ^&^*P* < 0.05 main effect of treatment by two-way ANOVA. ^∗^*P* < 0.05 and ^∗∗^*P* < 0.01.

To explore whether testosterone induces Nrf2 expression or promotes its nuclear translocation, Nrf2 and its downstream target HO-1 were detected by immunoblotting. Relative to WT-aged mice, significantly increased Nrf2 levels in the SN as well as in nucleus fraction were detected in WT-aged-TP mice (Figures 8(a), 8(b), 8(d), and 8(e)). Elevated HO-1 levels were shown in the SN of WT-aged-TP mice compared with WT-aged mice (*P* < 0.01), and increased HO-1 levels in the SN were not detected in KO-aged-TP mice relative to KO-aged mice. Supplementation with TP increased Nrf2 levels and promoted its nuclear translocation in WT-aged male mice.

### 3.9. The Effects of Nrf2 Deficiency on the Testosterone Efficiency in GDX Young Male Mice

**Figure 9:**
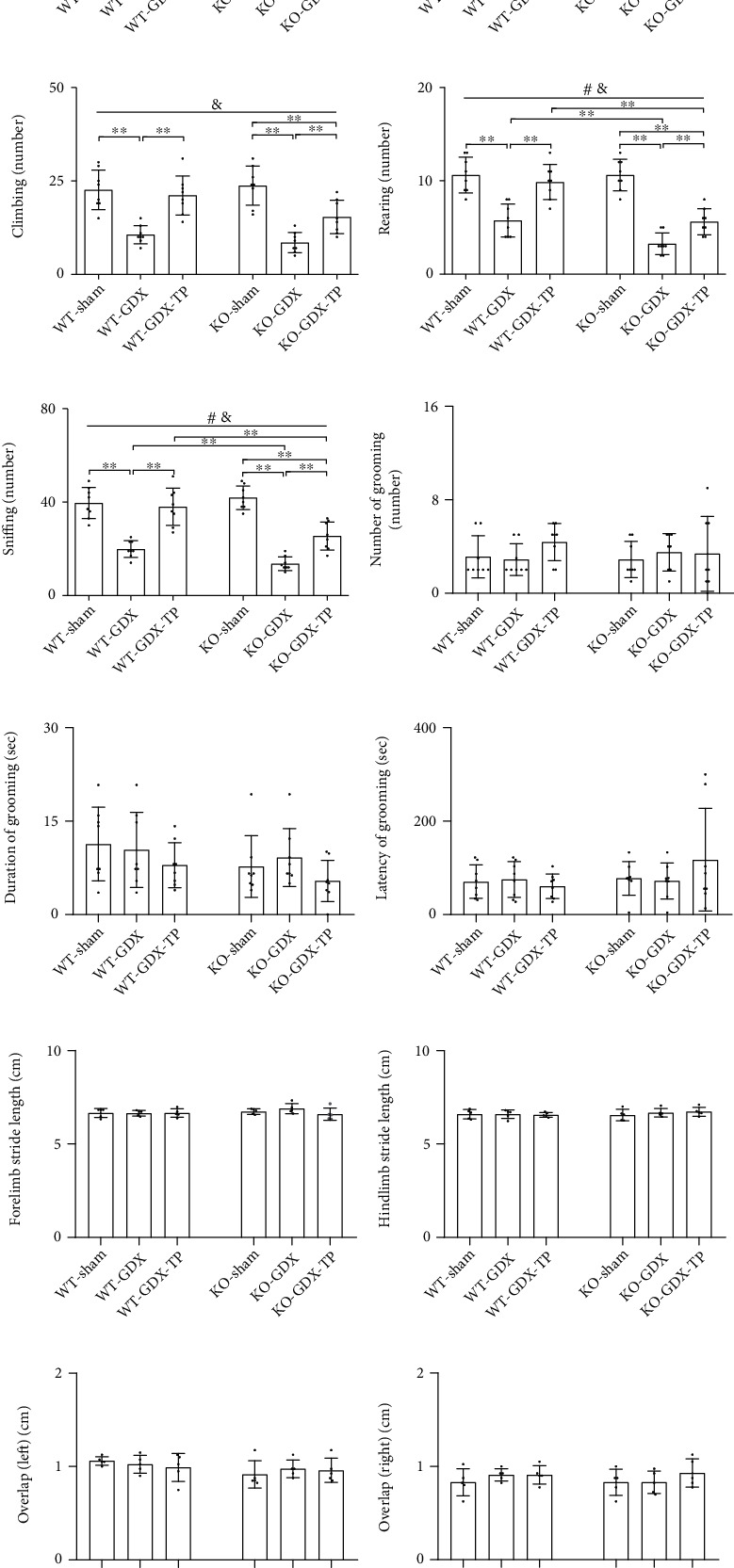
Effects of TP supplementation on open-field activity and walking gait of Nrf2 KO GDX male mice: (a) total path length, (b) walking, (c) climbing, (d) rearing, (e) sniffing, (f) number of grooming, (g) duration of grooming, (h) latency of grooming, (i) forelimb stride length, (j) hindlimb stride length, (k) overlap of left footprints, and (l) overlap of right footprints. Data were presented as mean ± SD; *n* = 8 for open-field test; *n* = 5 for footprint test. ^#^*P* < 0.05 main effect of genotype by two-way ANOVA; ^&^*P* < 0.05 main effect of treatment by two-way ANOVA. ^∗^*P* < 0.05 and ^∗∗^*P* < 0.01.

**Figure 10:**
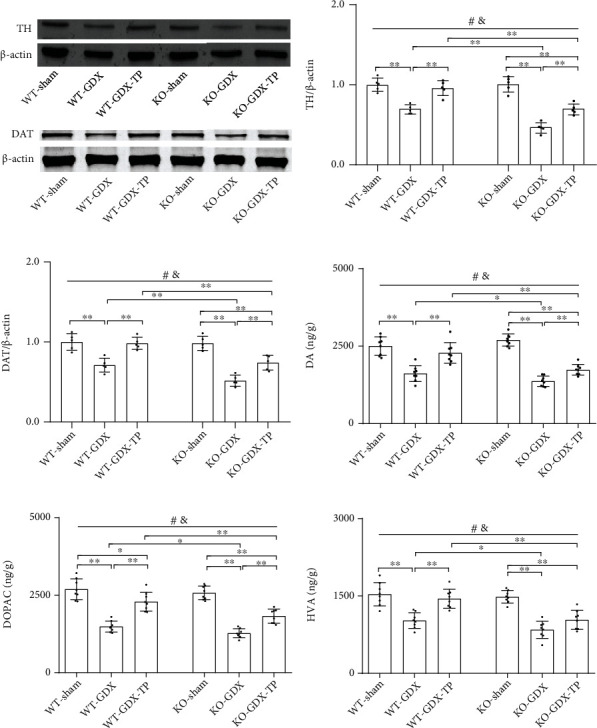
Effects of TP supplementation on dopaminergic activity in the caudate putamen of Nrf2 KO GDX male mice. (a, b) TH and (a, c) DAT were detected by immunoblotting. (d) DA, (e) DOPAC, and (f) HVA were measured by LC-MS/MS assay. Data were presented as mean ± SD; *n* = 5 for TH and DAT; *n* = 8 for DA, DOPAC, and HVA. ^#^*P* < 0.05 main effect of genotype by two-way ANOVA; ^&^*P* < 0.05 main effect of treatment by two-way ANOVA. ^∗^*P* < 0.05 and ^∗∗^*P* < 0.01.

**Figure 11:**
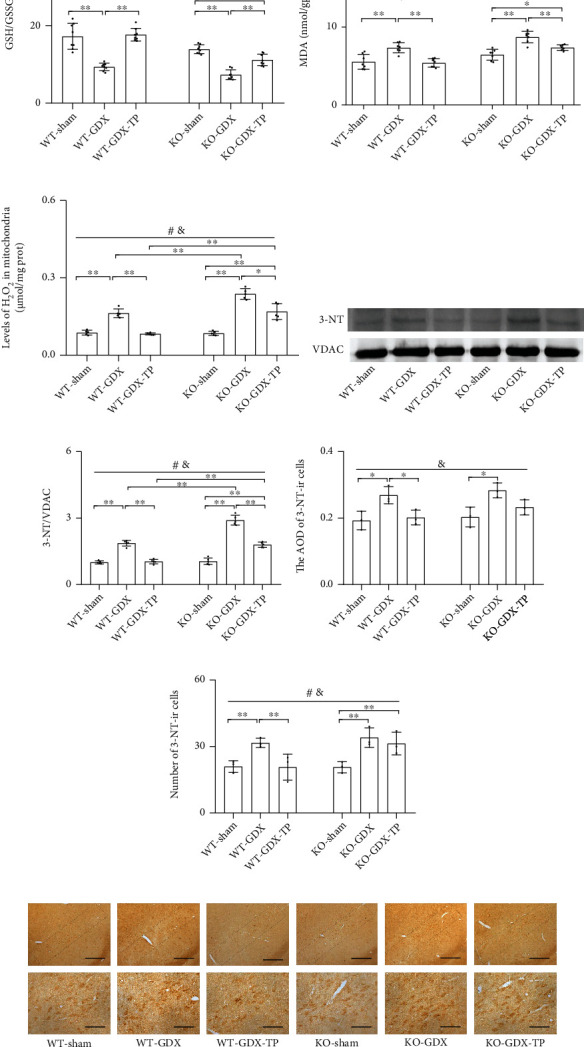
Effects of TP supplementation on oxidative balance in the substantia nigra of Nrf2 KO GDX male mice. (a) GSH/GSSG, (b) MDA, and (c) mitochondrial H_2_O_2_ were assessed by spectrophotometry. (d, e) Mitochondrial 3-NT was measured by immunoblotting; (f–h) 3-NT in the SN was detected by immunohistochemistry. Data were presented as mean ± SD; *n* = 8 for GSH/GSSG; *n* = 7 for MDA; *n* = 5 for mitochondrial 3-NT; *n* = 3 for 3-NT immunohistochemistry. Scale bars = 50 *μ*m (lower panel); scale bars = 200 *μ*m (upper panel). ^#^*P* < 0.05 main effect of genotype by two-way ANOVA; ^&^*P* < 0.05 main effect of treatment by two-way ANOVA. ^∗^*P* < 0.05 and ^∗∗^*P* < 0.01.

**Figure 12:**
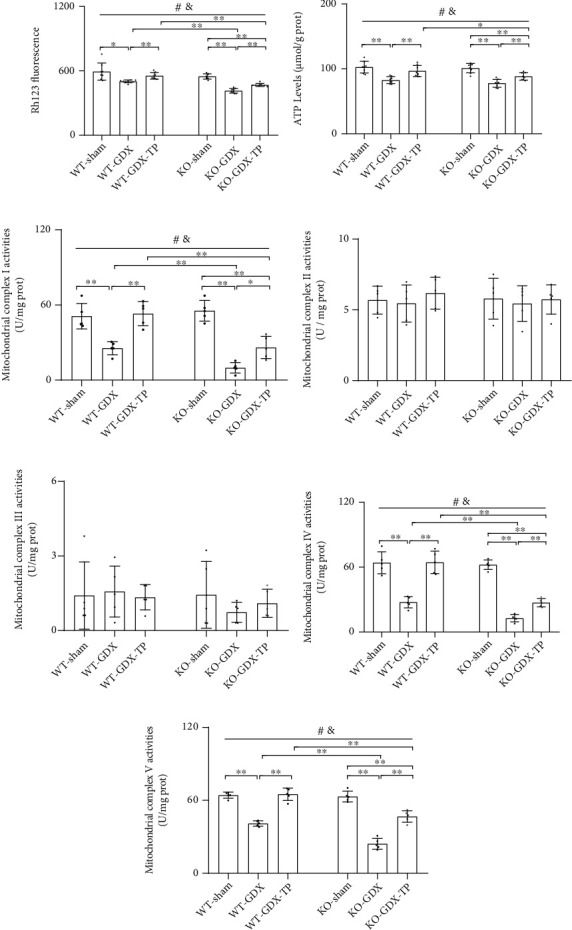
Effects of TP supplementation on mitochondrial function in the substantia nigra of Nrf2 KO GDX male mice. (a) Mitochondrial membrane potential was revealed using the Rh123 fluorescence method. (b) Mitochondrial ATP levels, (c) mitochondrial complex I activity, (d) mitochondrial complex II activity, (e) mitochondrial complex III activity, (f) mitochondrial complex IV activity, and (g) mitochondrial complex V activity were revealed by spectrophotometry. Data were presented as mean ± SD; *n* = 8 for MMP and mitochondrial ATP levels; *n* = 5 for mitochondrial complexes I-V. ^#^*P* < 0.05 main effect of genotype by two-way ANOVA; ^&^*P* < 0.05 main effect of treatment by two-way ANOVA. ^∗^*P* < 0.05 and ^∗∗^*P* < 0.01.

**Figure 13:**
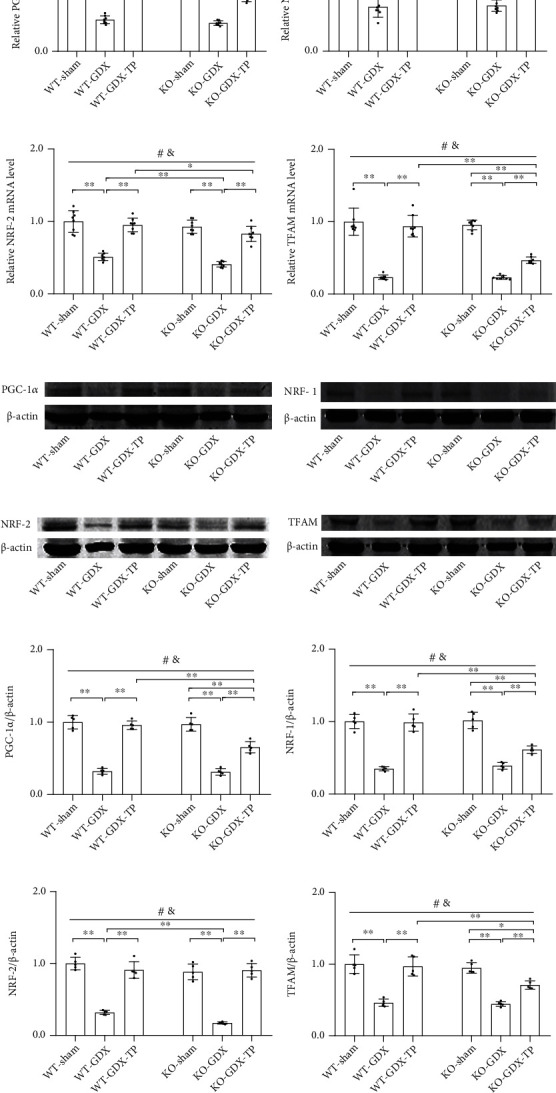
Effects of TP supplementation on mitochondrial biogenesis in the substantia nigra of Nrf2 KO GDX male mice. (a) *PGC-1α*, (b) *NRF-1*, (c) *NRF-2*, and (d) *TFAM* mRNA were revealed by qPCR. (e, i) PGC-1*α*, (f, j) NRF-1, (g, k) NRF-2, and (h, l) TFAM proteins were detected by immunoblotting. Data were presented as mean ± SD; *n* = 8 for qPCR; *n* = 5 for western blot. ^#^*P* < 0.05 main effect of genotype by two-way ANOVA; ^&^*P* < 0.05 main effect of treatment by two-way ANOVA. ^∗^*P* < 0.05 and ^∗∗^*P* < 0.01.

**Figure 14:**
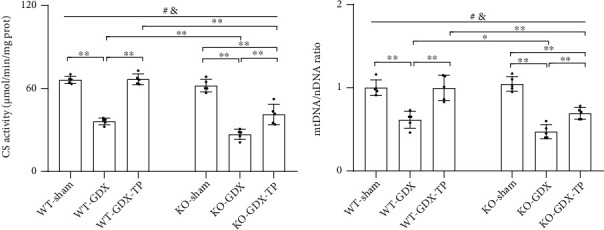
Effects of TP supplementation on mitochondrial content in the substantia nigra of Nrf2 KO GDX male mice. (a) CS activity was assessed by spectrophotometry. (b) mtDNA/nDNA was detected by qPCR. Data were presented as mean ± SD; *n* = 5. ^#^*P* < 0.05 main effect of genotype by two-way ANOVA; ^&^*P* < 0.05 main effect of treatment by two-way ANOVA. ^∗^*P* < 0.05 and ^∗∗^*P* < 0.01.

**Figure 15:**
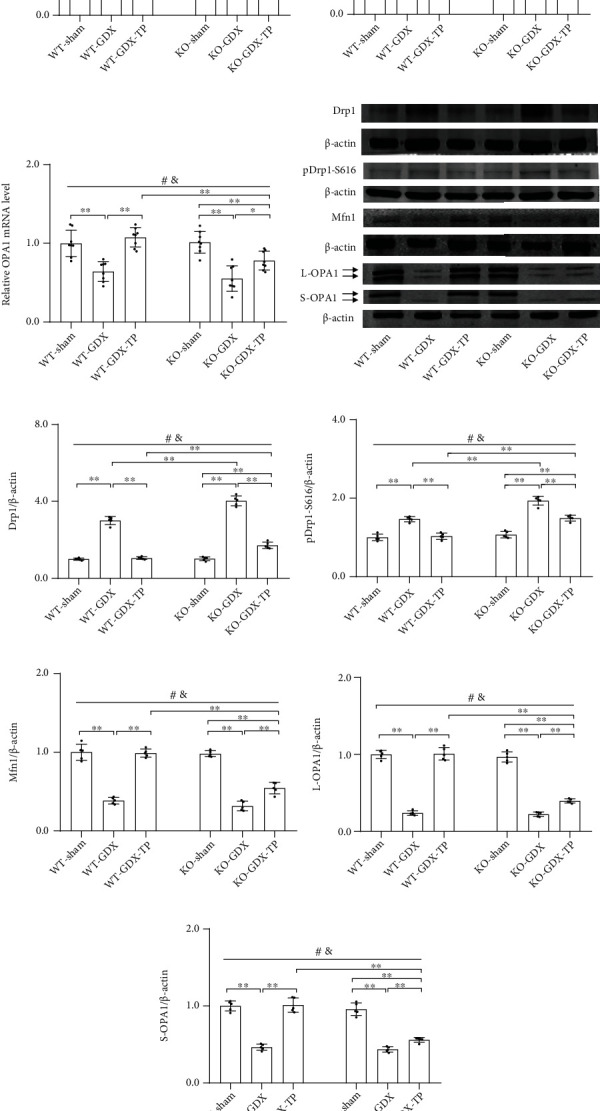
Effects of TP supplementation on mitochondrial dynamics in the substantia nigra of Nrf2 KO GDX male mice. (a) *Drp1*, (b) *Mfn1*, and (c) *OPA1* mRNA were revealed by qPCR. (d, e) Drp1, (d, f) pDrp1-S616, (d, g) Mfn1, (d, h) L-OPA1, and (d, i) S-OPA1 proteins were detected by immunoblotting. Data were presented as mean ± SD; *n* = 8 for qPCR; *n* = 5 for immunoblotting. ^#^*P* < 0.05 main effect of genotype by two-way ANOVA; ^&^*P* < 0.05 main effect of treatment by two-way ANOVA. ^∗^*P* < 0.05 and ^∗∗^*P* < 0.01.

Under the condition of ruling out aging factor, the above parameters detected in Experiment 1 were analyzed in the young experimental animal models of Experiment 2 (Figures 9–15). Orchiectomy to young mice of both genotypes weakened open-field activity (*P* < 0.01, Figures 9(a)–9(e)). Gonadectomized young male mice of both genotypes showed the decreased nigrostriatal dopaminergic activity (*P* < 0.01, Figures 10(a)–10(f)), the increased oxidative stress (GSH/GSSG, MDA, mitochondrial H_2_O_2_, and mitochondrial 3-NT, *P* < 0.01, Figures 11(a)–11(e). 3-NT: AOD, *P* < 0.05; number, *P* < 0.01, Figures 11(f)–11(h)), the reduced mitochondrial function (MMP: WT, *P* < 0.05; KO, *P* < 0.01, Figure 12(a). Mitochondrial ATP, mitochondrial complexes I, IV, and V, *P* < 0.01, Figures 12(b), 12(c), 12(f), and 12(g)), and the decreased expression levels of PGC-1*α*, NRF-1, NRF-2, and TFAM (*P* < 0.01, Figures 13(a)–13(l)), as well as the lowered mitochondrial content (CS, mtDNA/nDNA, *P* < 0.01, Figures 14(a) and 14(b)) in the studied brain region. The increased Drp1 and pDrp1-S616, as well as the reduced Mfn1, L-OPA1, and S-OPA1 levels, were found in the SN of gonadectomized young male mice of both genotypes (*P* < 0.01, Figures 15(a)–15(i)). KO-GDX mice presented the aggravated effects of orchiectomy on the above parameters compared with WT-GDX mice, except for climbing (Figure 9(c)), 3-NT (AOD and number of 3-NT-ir cells, Figures 11(f)–11(h)), ATP (Figure 12(b)), PGC-1*α* (Figures 13(a), 13(e), and 13(i)), NRF-1 (Figures 13(b), 13(f), and 13(j)), TFAM (Figures 13(d), 13(h), and 13(l)), Mfn1 (Figures 15(b), 15(d), and 15(g)), OPA1 mRNA (Figure 15(c)), L-OPA1 (Figures 15(d) and 15(h)), and S-OPA1 (Figures 15(d) an(d) 15(i)). Supplementation with TP restored the observed parameters above in WT-GDX mice to WT-sham level except for DOPAC (*P* < 0.05, Figure 10(e)). The above parameters in KO-GDX-TP mice were ameliorated except for HVA (Figure 10(f)) and 3-NT (AOD and number of 3-NT-ir cells, Figures 11(f)–11(h)) relative to KO-GDX mice, but not reversed to KO-sham level except for NRF-2 (Figures 13(c), 13(g), and 13(k)). NRF-2 level in the SN of KO-GDX-TP mice reached the level of KO-sham mice.

## 4. Discussion

In this study, we demonstrated that WT-aged male mice presented uncoordinated walking gait, as well as declined open-field activity, nigrostriatal dopaminergic activity, oxidative balance, and mitochondrial function, as well as downregulated mitochondrial biogenesis and mitochondrial dynamics. Nrf2 deficiency exacerbated the deficits of the above parameters in aged male mice. Supplementation with testosterone to WT-aged male mice significantly ameliorated open-field activity, walking gait, nigrostriatal dopaminergic activity, oxidative balance, and mitochondrial function and upregulated mitochondrial biogenesis and mitochondrial dynamics. However, the above effects of testosterone on WT-aged male mice were not shown in Nrf2 KO-aged male mice. Orchidectomy to young male mice decreased mitochondrial function, downregulated mitochondrial biogenesis, and altered mitochondrial dynamics balance. Supplementation with testosterone to Nrf2 KO-GDX mice only ameliorated the alterations above but did not reverse them to sham level. Thus, Nrf2 plays an important role in ameliorating open-field activity, walking gait, and nigrostriatal dopaminergic activity of aged male mice by testosterone supplementation, which is related to the activated Nrf2 by TP. Nrf2 deficiency attenuated the efficiency of testosterone supplementation in improving mitochondrial function of the substantia nigra in aged male mice via mitochondrial biogenesis and mitochondrial dynamics to some extent.

Motor activity gradually declines during the aging process [29], and this decline is related to the age-related decline of nigral neuronal function [29, 31]. A persistent loss of nigrostriatal neurons results in severe locomotor impairment, such as altered gait pattern [32]. Previous studies demonstrated that androgen influences brain function to induce behavioral alterations [14, 33]. Androgen supplementation increases motor and exploratory behaviors of aged male rodents [14, 18, 33] and ameliorated motor symptoms of men with PD to some extent [34]. The amelioration of motor and exploratory behaviors in testosterone-supplemented aged animals is related to nigrostriatal dopaminergic activity enhanced by androgen [14, 15]. The present study showed that supplementation with testosterone significantly improved motor and exploratory behaviors, as well as walking gait of WT-aged male mice and enhanced nigrostriatal dopaminergic activity. However, the above effects of testosterone supplementation on WT-aged male mice were not found in Nrf2 KO-aged male mice. The administration of testosterone to Nrf2 KO-aged male mice neither improved motor and exploratory behaviors as well as walking gait nor enhanced nigrostriatal dopaminergic activity, which demonstrated that Nrf2 might be required for testosterone to ameliorate their neurochemical defects of the nigrostriatal dopaminergic system in aged male mice. Nrf2 is widely expressed in the central nervous system including the SN. As a master transcription factor, Nrf2 binds ARE in the promoters of Nrf2 target genes [9, 35]. When it is activated, a series of cytoprotective enzymes and antioxidants are induced [35], such as HO-1. Nrf2 with its downstream gene products constitutes the main antioxidant defense system to degrade free radicals [9, 35]. The previous studies showed that Nrf2 protects nigrostriatal dopaminergic neurons and maintains their normal activity [9]. In PD, remaining dopaminergic neurons exhibit stronger nuclear Nrf2 immunoreactive staining [36]. Thus, our results above indicated that Nrf2 might determine the ameliorative effects of testosterone supplementation on the nigrostriatal dopaminergic system of aged male mice.

How does testosterone exerts its ameliorative effects on the nigrostriatal dopaminergic system of aged male animals has not been elucidated. Improved mitochondrial function might explain the ameliorative effects of TP supplementation on the nigrostriatal dopaminergic system during aging process by modulating oxidative stress, mitochondrial biogenesis, and mitochondrial dynamics. Oxidative stress is characterized by an unbalance between overproduced ROS and antioxidant defenses in cells. It is one of the major factors in aging and in aging-related neurodegenerative diseases [37]. Under physiological conditions, cellular ROS are eradicated by the endogenous antioxidative defense systems. However, under pathological circumstances, mitochondrial dysfunction, such as reduced MMP (an important indicator of mitochondrial function) and impaired oxidative phosphorylation, produces excessive ROS, resulting in oxidative damage to cells by targeting proteins, lipids, or DNA [2]. The mitochondrion is a main organelle producing superoxide anion (O_2_^−^). It is involved in oxidative damage to macromolecules and can be converted to O_2_ and H_2_O_2_. GSH and GSSG are two different forms of glutathione. Glutathione peroxidase catalyzes the reduction of H_2_O_2_ via GSH to produce GSSG and H_2_O. MDA is an important indicator for detecting lipid peroxidation of biological membranes. It is the peroxidation product of phospholipids or lipoproteins of cytoplasmic membranes [38]. 3-NT is another biomarker of oxidative stress to predict the level of oxidative damage [39, 40]. It is formed due to nitration of protein-bound and free tyrosine residues by reactive peroxynitrite molecules. The previous study showed that the levels of Nrf2 and its downstream gene products, such as NQO-1 and HO-1, are higher in TP-treated aged male rats than in their counterpart control [15]. TP-treated aged male rats show decreased MDA and GSH/GSSG in the SN compared with control [15, 18]. In the present study, decreased GSH/GSSG and increased MDA as well as mitochondrial H_2_O_2_ and 3-NT revealed the existed oxidative damage in the SN of aged male mice, especially in Nrf2 KO-aged male mice. Decreased MMP and mitochondrial ATP levels, as well as activities of mitochondrial complexes I, IV, and V, indicated mitochondrial dysfunction in the SN of aged male mice. Testosterone supplementation decreased MDA, as well as mitochondrial H_2_O_2_ and 3-NT levels, and increased GSH/GSSG ratio, MMP, mitochondrial ATP content, and activities of mitochondrial complexes I, IV, and V in the SN of WT-aged male mice. The results above showed improved mitochondrial function in WT TP-treated aged male mice. By comparing the results of 3-NT immunohistochemistry with those of 3-NT immunoblotting, it was found that although both methods detected a significant increase in 3-NT levels in the SN of WT-aged male mice before TP treatment, immunohistochemistry-revealed 3-NT levels only showed decreased trend in WT-aged-TP mice, not reaching significance. However, immunoblotting detected significantly decreased 3-NT levels in WT-aged-TP mice. The former observed the cells in the SN, while the latter located isolated mitochondria from the SN, which will more accurately reflect the subtle changes in damaged organelles. Mitochondria, as primary sources of ROS, were damaged in WT-aged male mice. The mitochondrial ultrastructure by an electron microscope supported the finding above. WT-aged mice showed ultrastructural abnormalities of mitochondria in a way, such as unclear cristae, which was improved by TP supplementation. However, improved oxidative balance status and mitochondrial function were only shown in the SN of TP-treated WT-aged male mice, not in TP-treated Nrf2 KO-aged male mice. It was suggested that Nrf2 deficiency blocked the efficiency of testosterone replacement in ameliorating mitochondrial function in the SN of aged male mice, leading to oxidative damage to SN.

Aging and aging-related neurodegenerative disorders are often accompanied by mitochondrial dysfunction [21]. During aging process, promoting mitochondrial biogenesis might be a cellular strategy to maintain normal mitochondrial function [6]. Mitochondrial biogenesis is mainly regulated by PGC-1*α*, a key regulator of mitochondrial biogenesis and antioxidative defense [8, 41]. It controls the rate of the mitochondrial biogenesis through its downstream targets, NRF-1, NRF-2, and TFAM [41, 42]. *In vitro* study showed that testosterone increases PGC-1*α*, NRF-1, and TFAM at transcription level in C_2_C_12_ myotubes [43, 44] and flutamide (androgen receptor antagonist) reduces the testosterone-induced upregulation of PGC-1*α* [44], NRF-1, and TFAM [43]. Moreover, testosterone deficiency caused by orchiectomy decreases PGC-1*α*, NRF-1, and TFAM gene expression in the adult rat hippocampus and supplementation of testosterone to orchiectomy adult rats restores them in the hippocampus to control levels [19]. Similarly, we found in the present study that orchiectomy reduced PGC-1*α*, NRF-1, NRF-2, and TFAM gene expression in the SN of WT-young male mice. Reduced PGC-1*α*, NRF-1, NRF-2, and TFAM gene expression, with decreased CS activity and mtDNA copy number in the SN of GDX WT-young male mice, was reversed to sham levels by TP supplementation, which suggested that mitochondrial biogenesis in the SN is androgen-related. Mitochondrial biogenesis has been found to decline in the skeletal muscle and in the brain during aging [18, 45]. During the aging process, PGC-1*α* expression level declines in the skeletal muscle as well as in the brain and this can be reversed in the skeletal muscles by exercise training of aged male rats [46] and ameliorated in the brain by TP supplementation to aged male rats [18], which demonstrated the aging skeletal muscle cells and neurons still remain the ability to upregulate mitochondrial biogenesis through increasing PGC-1*α* expression [18, 46]. Narasimhan et al. found that the PGC-1*α* protein levels were decreased in the skeletal muscle of Nrf2 KO-aged mice compared with WT-aged mice [47]. In the present study, we found that PGC-1*α* and its downstream target expression levels in the SN were significantly reduced in WT-aged mice and Nrf2 KO further decreased their expression levels in the SN upon aging. In addition, CS activity, mtDNA copy number, and mitochondrial number were significantly reduced in the SN of KO-aged mice due to Nrf2 deficiency. mtDNA copy number is frequently used as an index of mitochondrial content. CS activity is a more accurate indicator for mitochondrial content in tissues than mtDNA copy number [48]. Mitochondrial number in cells by electron microscopy directly displays the status of mitochondrial biogenesis and mitochondrial content. Supplementation with TP to WT-aged mice increased expression of PGC-1*α* and its downstream targets, as well as CS activity, mtDNA copy number, and mitochondrial number in the SN, and such effects of TP supplementation on them in the SN were blocked in Nrf2 KO-aged mice due to Nrf2 deficiency. A previous *in vitro* study revealed that silencing the PGC-1*α* gene in SH-SY5Y cells results in the reduction of MMP and intracellular ATP content as well as the elevation of intracellular H_2_O_2_ generation [49]. Thus, the improvement of mitochondrial function (i.e., increased MMP, mitochondrial ATP, and complex activities, as well as decreased MDA, mitochondrial H_2_O_2_, and 3-NT and increased GSH/GSSG) in the SN of TP-treated WT-aged mice was associated with the TP-promoted mitochondrial biogenesis in large extent. Nrf2 played an important role in the effects of testosterone supplementation on the SN mitochondrial biogenesis of aged male mice.

The balanced mitochondrial dynamics is another important factor for improving mitochondrial function in the maintenance of normal cell activity [50–52]. Mitochondria undergo continuous rounds of fusion and fission in their life and form into dynamic networks within cells. Balanced mitochondrial dynamics are regulated by the GTPase family of proteins including Drp1, Mfn1/Mfn2, and OPA1 [53–55]. Drp1 is responsible for the mitochondrial fission [53]. Drp1 activity is regulated by its phosphorylation. Phosphorylation at Ser616 enhances Drp1-mediated fission [56]. Mfn1/Mfn2 and OPA1 are involved in the mitochondrial fusion [54, 55]. OPA1 activity is regulated by L-OPA1 and S-OPA1 through OPA1 cleavage. Both L-OPA1 and S-OPA1 are necessary for mitochondrial fusion; however, L-OPA1 or S-OPA1 alone is not sufficient for fusion under normal conditions [57]. The expression of Drp1 and OPA1 seemed to be affected by androgen levels. In an androgenized rat model for polycystic ovary syndrome, androgen induces upregulation of Drp1 [58]. Treatment of androgen-sensitive prostate cancer cells with an androgen receptor agonist or antagonist revealed that Drp1 is transcriptionally regulated by them [59]. In addition, it was found that testosterone increases fusion protein OPA1 expression in C_2_C_12_ cells [43]. In the present study, we found that testosterone deficiency induced by orchiectomy disrupted balanced mitochondrial dynamics in the SN of WT young male mice. Orchiectomy increased Drp1 and pDrp1-S616 levels and decreased Mfn1, L-OPA1, and S-OPA1 levels in the SN of WT young mice. It was indicated that there might be more fragmented mitochondria in the SN of GDX young male mice, which affects mitochondrial function and metabolism [51]. Unlike GDX young male mice, aged male mice showed the decreased levels of Drp1, pDrp1-S616, Mfn1, L-OPA1, and S-OPA1 in the SN. The difference related to mitochondrial dynamics between aged male mice and GDX young male mice showed that the ability of both mitochondrial fusion and mitochondrial fission was downregulated in aged male mice. Previous studies revealed the dysregulated mitochondrial dynamics in aging-related neurodegenerative diseases [60, 61]. Specifically, increased mitochondrial fission and reduced mitochondrial fusion are major features in aging-related neurodegenerative diseases [60, 62]. However, in contrast with the different expression state between fission proteins and fusion proteins in pathological conditions, natural aging shows the declined expression of both fission proteins and fusion proteins [63, 64]. Mitochondrial fission-fusion mRNA and protein expression levels are differentially altered in the aging process, but they reach significantly lower levels of mitochondrial dynamics proteins in older animals [64]. Decreased Drp1 was found in several aged mouse tissues including neurons, in cultured aged human endothelial cells, or in old human skeletal muscle [63–65], and reduced Mfn1 and OPA1 were also detected in the skeletal muscle of aged mice [64, 65]. The balance between mitochondrial fusion and fission is clearly crucial for neuronal function [51]. Therefore, aged organisms might try to maintain a balanced mitochondrial dynamics through downregulating both fission and fusion protein levels. However, altered mitochondrial dynamics by downregulating mitochondrial dynamics proteins in the aging process might not be efficient enough to maintain normal mitochondrial structures and functions in aged animals [65, 66] as young animals do. Supplementation with TP upregulated Drp1 and pDrp1-S616 involved in fission, as well as Mfn1, L-OPA1, and S-OPA1 related to fusion in the SN of WT-aged male mice, thus ameliorating their mitochondrial dynamics in the SN. However, this effect of TP supplementation in WT-aged mice was not detected in Nrf2 KO-aged male mice. Nrf2 deficiency blocked efficiency of TP supplementation in aged male mice. The present results indicated that the regulation of mitochondrial dynamics in aged male mice was affected by testosterone levels and Nrf2 was a key factor for TP upregulation of mitochondrial dynamics in the SN of aged male mice. Mitochondrial fusion contributes to increasing mitochondrial ATP production [67]; therefore, the increased ATP levels, as well as improved status of nigrostriatal dopaminergic neurons in the SN of TP-treated WT-aged male mice, suggested that the final net effects of altered mitochondrial dynamics by TP supplementation might promote mitochondrial fusion, which should be testified in the future studies.

Both testosterone level declines and age-related mitochondrial dysfunction are present in aging men and rodents [18, 46, 68]. Coincidentally, Nrf2 also decreases during aging process [69]. It seemed that there was close association among them. An age-related decline in Nrf2 function might play an important role between loss of testosterone and declined mitochondrial function during aging process. It was found that Nrf2 is involved in the maintenance of mitochondrial function through modulating cellular redox balance and MMP [70]. Activation of Nrf2 would contribute to the improved mitochondrial function during aging process. In the present study, we found that supplementation with TP increased Nrf2, as well as its downstream target HO-1 expression, and promoted Nrf2 nuclear translocation in the aged male mice. Thus, loss of testosterone during aging process might reduce activation of Nrf2, affecting mitochondrial function. Ameliorated mitochondrial function in the SN of WT-aged-TP mice was related to Nrf2 activation by TP supplementation to large extent. Nrf2 deficiency exacerbated mitochondrial dysfunction in the SN of aging male mice, and TP supplementation did not ameliorate the exacerbated mitochondrial dysfunction in KO-aged male mice. The following might explain the difference of mitochondrial function in the SN of TP-supplemented aged male mice of both genotypes. One was direct involvement of Nrf2 in the regulation of mitochondrial function in this process. The study by Piantadosi et al. checked for Nrf2-binding sites in the NRF-1 promoter and found four AREs in the NRF-1 gene promoter [71]. Nrf2 binds to AREs of NRF-1 promoter, activates NRF-1, and induces mitochondrial biogenesis via TFAM [71]. TP supplementation to aged male mice increased nuclear Nrf2 levels in the SN, thus improving mitochondrial function of aged male mice through promoting mitochondrial biogenesis. This effect of TP supplementation was significantly blocked due to Nrf2 knockout. Another was the oxidative stress milieus of cells, whether it was ameliorated by the regulation of antioxidants and antioxidant gene expression. The oxidative stress status among experimental groups was different in the present study. Significantly increased oxidative stress was present in KO-aged mice, as well as KO-aged-TP mice, compared with WT-aged mice. Testosterone has protective effects in a low oxidative stress environment [22]. And these protective effects will be weakened or even harmful in the state of high oxidative stress [22, 72]. In addition, by comparing the studies on castration and androgen replacement in young male mice, we also found that the effects of TP supplementation were significantly weaker in KO-GDX mice than WT-GDX mice, and the oxidative stress state of the SN was significantly lower in WT-GDX mice than KO-GDX mice. Thus, the present results that Nrf2 deficiency attenuated the efficiency of TP supplementation was to a certain extent due to high oxidative stress state. Activation of Nrf2 to reduce oxidative stress was beneficial for testosterone to play its ameliorative effects on mitochondrial dysfunction in aging and aged-related neurodegenerative disorders in males. Considering the involvement of Nrf2 in regulating mitochondrial functions, it was presumed that Nrf2 might also affect mitochondrial dynamics through direct or indirect way in TP supplementation to aged male mice. However, direct experimental evidence for Nrf2 roles in mitochondrial dynamics is limited [11]. So, it was unclear to what extent changes in mitochondrial dynamics in the present studies were involved in the improvement of mitochondrial function by TP. The changed mitochondrial dynamics in TP-treated aged male mice was causal in improved mitochondrial function, or merely a downstream consequence should be further clarified in the future study.

## 5. Conclusion

In summary, Nrf2 knockout further reduced the nigrostriatal dopaminergic neurochemical content and aggravated downregulation of both mitochondrial biogenesis and mitochondrial dynamics in the substantia nigra upon aging. Supplementation with testosterone induced the amelioration on nigrostriatal dopaminergic activity and mitochondrial function in aged male animals by modulation of mitochondrial biogenesis and mitochondrial dynamics. Nrf2 deficiency blocked testosterone-induced upregulation of mitochondrial biogenesis and mitochondrial dynamics in the aging substantia nigra. Thus, as a target, activation of Nrf2 might be conducive to testosterone-upregulating mitochondrial biogenesis and mitochondrial dynamics in the substantia nigra upon aging to produce mitochondria that are more efficient in ATP production and have optimal oxidative capacity.

## Data Availability

The data that support the findings of this study are available from the corresponding author upon request.

## Supplementary Materials

Supplementary Materials Table S1: accession numbers of the genes for primers.
