# Supplementary material for: Nrf2 Deficiency Attenuates Testosterone Efficiency in Ameliorating Mitochondrial Function of the Substantia Nigra in Aged Male Mice
Source: Oxid Med Cell Longev. 2022 Feb 18;2022:3644318. doi: 10.1155/2022/3644318 (PMC8881137; doi:10.1155/2022/3644318)
Supplement: Supplementary Materials — Table S1: accession numbers of the genes for primers. [file 3644318.f1.doc]

TABLE S1: Accession numbers of the genes for primers.

| Gene | Accession numbers |
| --- | --- |
| *PGC-1α* | [JX866948.1](https://www.ncbi.nlm.nih.gov/nucleotide/JX866948.1?report=genbank&log$=nucltop&blast_rank=2&RID=P0090A37016) |
| *NRF-1* | [BC005410.1](https://www.ncbi.nlm.nih.gov/nucleotide/BC005410.1?report=genbank&log$=nucltop&blast_rank=23&RID=P00WFHFV016) |
| *NRF-2* | [AH006764.2](https://www.ncbi.nlm.nih.gov/nucleotide/AH006764.2?report=genbank&log$=nucltop&blast_rank=1&RID=P010UY46013) |
| *TFAM* | [BC083084.1](https://www.ncbi.nlm.nih.gov/nucleotide/BC083084.1?report=genbank&log$=nucltop&blast_rank=4&RID=P0130PD5013) |
| *Drp1* | [BC040777.1](https://www.ncbi.nlm.nih.gov/nucleotide/BC040777.1?report=genbank&log$=nucltop&blast_rank=30&RID=P018MD1K016) |
| *Mfn1* | [BC056641.1](https://www.ncbi.nlm.nih.gov/nucleotide/BC056641.1?report=genbank&log$=nucltop&blast_rank=4&RID=P01B82FU016) |
| *OPA1* | [BC145959.1](https://www.ncbi.nlm.nih.gov/nucleotide/BC145959.1?report=genbank&log$=nucltop&blast_rank=9&RID=P01E1PH9013) |
| *GAPDH* | GU214026.1 |
| *16S rRNA (mtDNA)* | LC062083.1 |
| *HK2 (nDNA)* | JN957201.1 |
